# Association between weight-bearing ankle dorsiflexion range of motion during deep squat sitting and quality of life after ankle fracture surgery: a cross-sectional study

**DOI:** 10.3389/fresc.2025.1645621

**Published:** 2025-08-04

**Authors:** Hayato Miyasaka, Bungo Ebihara, Takashi Fukaya, Koichi Iwai, Shigeki Kubota, Hirotaka Mutsuzaki

**Affiliations:** ^1^ Department of Rehabilitation, Tsuchiura Kyodo General Hospital, Tsuchiura, Japan; ^2^ Graduate School of Health Sciences, Ibaraki Prefectural University of Health Sciences, Ami, Japan; ^3^ Department of Physical Therapy, Faculty of Health Sciences, Tsukuba International University, Tsuchiura, Japan; ^4^ Center for Humanities and Sciences, Ibaraki Prefectural University of Health Sciences, Ami, Japan; ^5^ Department of Occupational Therapy, School of Health Sciences, Ibaraki Prefectural University of Health Sciences, Ami, Japan; ^6^ Center for Medical Science, Ibaraki Prefectural University of Health Sciences, Ami, Japan; ^7^ Department of Orthopedic Surgery, Ibaraki Prefectural University of Health Sciences Hospital, Ami, Japan

**Keywords:** ankle fracture, quality of life, self-administered foot evaluation questionnaire, ankle dorsiflexion, range of motion, deep squatting, lunge, walking

## Abstract

**Background:**

Ankle fracture is a common type of trauma. Although ankle fractures reduce the quality of life (QOL), few studies have investigated this factor, and even fewer have investigated the impact of postoperative physical function on reduced QOL. We aimed to clarify the physical factors that affect the QOL after ankle fracture surgery.

**Methods:**

This cross-sectional study included 32 patients who underwent surgery for ankle fractures. QOL was assessed using the Self-Administered Foot Evaluation Questionnaire (SAFE-Q). Ankle dorsiflexion range of motion (ROM) was measured with and without weight bearing. The weight-bearing ankle dorsiflexion ROM was measured using four methods: measuring the rear ankle with the knee extended and flexed, measuring the front ankle, and measuring the ankle during deep squat sitting. Gait parameters were measured using a three-dimensional motion analyzer. Multivariate analysis was performed using the four subscales of the SAFE-Q (pain and pain-related, physical functioning and daily living, social functioning, and general health and well-being) as dependent variables.

**Results:**

The multivariate analysis revealed that weight-bearing ankle dorsiflexion ROM during deep squat sitting was an independent variable for pain and pain-related [standardized partial regression coefficient (*β*) = 0.584, *P* < 0.001], physical functioning and daily living (*β* = 0.376; *P* = 0.006), social functioning (*β* = 0.317; *P* = 0.045), and general health and well-being (*β* = 0.483; *P* = 0.005). Gait speed was selected as an independent variable for physical functioning and daily living (*β* = 0.555; *P* < 0.001) and social functioning (*β* = 0.514; *P* = 0.002).

**Conclusions:**

Weight-bearing ankle dorsiflexion ROM during deep squat sitting and gait speed were associated with QOL of patients after ankle fracture surgery. These findings may inform treatment programs to improve QOL after ankle fractures and provide the theoretical background necessary for the development of new treatments.

## 1 Introduction

Ankle fractures are common lower limb fractures, accounting for approximately 9% of all fractures (1, 2). They reduce quality of life (QOL) (3). QOL captures the concept of health as defined by the World Health Organization, and a decline in QOL may negatively impact successful aging (4, 5). Therefore, postoperative QOL is an important outcome for patients with ankle fractures. However, only a limited number of studies have investigated factors related to postoperative QOL in the field of ankle fractures (6). Lorente et al. (7) reported age, sex, diabetes, body mass index (BMI), and smoking as factors affecting QOL; however, they did not consider variables related to physical function that may affect QOL. After ankle fracture surgery, patients experience persistently reduced physical function, including range of motion (ROM), muscle strength, and gait parameters (8–10). Therefore, the factors affecting QOL, including the above variables, need to be comprehensively characterized by considering them simultaneously. Although previous studies have evaluated general ROM for the ankle or gait parameters following ankle fractures (8–10), none have specifically examined how ankle dorsiflexion ROM, under high-demand, weight-bearing conditions, such as deep squat sitting, affects multiple domains of QOL.

One of the methods for evaluating QOL related to the ankle is the Self-Administered Foot Evaluation Questionnaire (SAFE-Q) (11). SAFE-Q is a patient-reported outcome measure proven to have sufficient reliability and validity (11). SAFE-Q is composed of six subscales (pain and pain-related, physical functioning and daily living, social functioning, shoe-related, general health and well-being, and sports activity) and allows evaluation reflecting the Asian lifestyle, such as deep squat sitting (11). In daily life, most movements are performed under load, such as standing and sitting, gait, stair climbing, and deep squat sitting, and ROM under load may have a greater impact on QOL than non-load-bearing; however, the relationship between these are unclear. Furthermore, variations exist in the measurement of weight-bearing ankle dorsiflexion ROM. However, the measurement method most relevant to QOL has not yet been identified.

This study aimed to clarify the physical factors related to the QOL of patients after ankle fracture surgery, with particular attention to SAFE-Q subscales that are more likely to decline after surgery: pain and pain-related, physical functioning and daily living, social functioning, and general health and well-being. We hypothesized that weight-bearing ankle dorsiflexion ROM is associated with postoperative QOL. In particular, we hypothesized that ankle dorsiflexion ROM during deep squat sitting would affect postoperative QOL. This study may facilitate the planning and development of new evidence-based rehabilitation programs for patients after ankle fracture surgery by identifying the effects of weight-bearing dorsiflexion ROM on postoperative QOL.

## 2 Materials and methods

### 2.1 Study design

This cross-sectional, observational study included patients who underwent ankle fracture surgery. The ethics committee of our institute approved this study, which was conducted in accordance with the principles of the Declaration of Helsinki. All participants provided written informed consent before participation.

### 2.2 Participants

The study was conducted between July 2022 and November 2024 and enrolled patients with ankle fractures admitted to our hospital. The inclusion criteria were ankle fractures treated with open surgery and physical therapy. All patients underwent open reduction and internal fixation (ORIF) and were immobilized with a splint for at least 1 week postoperatively. The exclusion criteria included multiple fractures, open fractures, postoperative complications, such as infection or deep vein thrombosis, history of neurological and orthopedic diseases, refusal to undergo postoperative measurements, and visits to another hospital. Participants were assessed 3 months after ORIF. All measurements were performed by the same physiotherapist. All participants used crutches for at least the first 3 weeks and continued a training program for joint mobility, muscle strength, and functional skills, such as walking and stair climbing, at least once a week for 3 months. The data on participants' age, sex, height, number of fractures (12, 13), and Lauge–Hansen classification (14) were collected from medical records. Participants' weights were measured using a digital scale, and their BMIs were calculated.

### 2.3 SAFE-Q measurement

The SAFE-Q was used to measure ankle QOL. It consists of 43 questions across 6 subscales: 9 questions on pain and pain-related (Q1–Q7, Q10, and Q11), 11 on physical functioning and daily living (Q12–Q22), 6 on social functioning (Q23–Q28), 3 on shoe-related (Q8–Q9, Q34), 5 on general health and well-being (Q29–Q33), and 9 assessing sports activity (Q35–Q43). The patients answered each question on a Likert scale (4, 3, 2, 1, or 0). Questions 3 and 43 were scored using a visual analog scale, with the formula (10−value) × 0.4 used to calculate the score. The score for each subscale was calculated as follows (11, 15):

(∑ofsubscale×25/numberofsubscalequestions)/100,

with higher subscale scores indicating higher QOL (16).

Each participant completed the questionnaire, and the examiners collected the data. In this study, 4 subscales were tabulated and scored on a maximum 100-point scale: pain and pain-related, physical functioning and daily living, social functioning, and general health and well-being.

### 2.4 Measurement of non-weight-bearing ankle ROM

Non-weight-bearing ankle ROM was measured in 1° increments using a goniometer during passive movement. With the participants in the supine position, ankle dorsiflexion ROM was measured with the knee in extended and flexed positions, and plantarflexion ROM was measured with the knee in flexed position. To measure passive ROM, the examiner manually dorsiflexed and plantarflexed the participant's ankle maximally. The examiner set the axis perpendicular to the fibula as the cardinal axis and the plantar surface of the foot as the axis of movement, and the angle between these axes was measured as ankle dorsiflexion and plantarflexion ROMs.

### 2.5 Measurement of weight-bearing ankle dorsiflexion ROM

**Figure 1:**
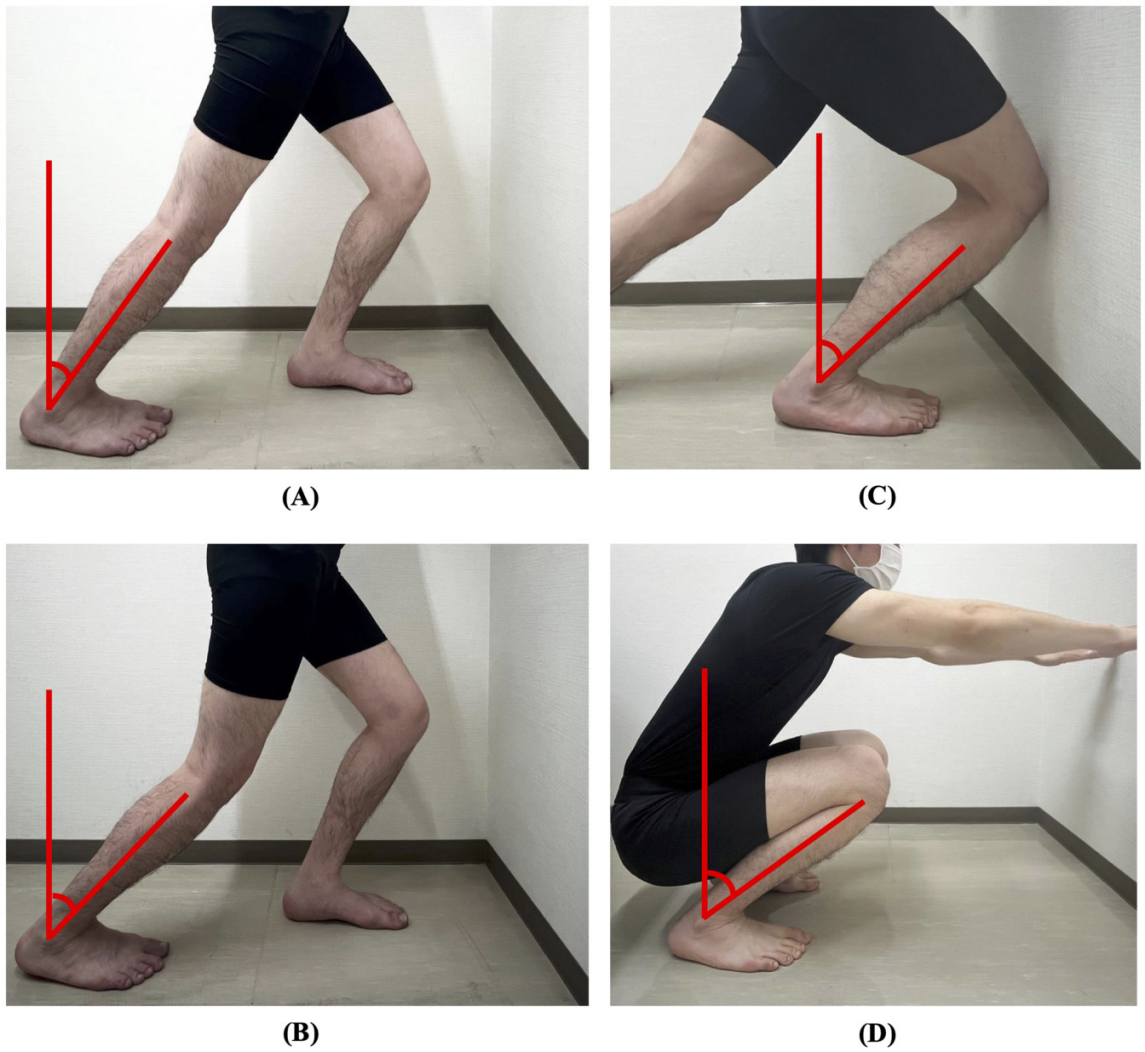
Weight-bearing ankle dorsiflexion range of motion measurement. **(A)** Rear ankle with the knee extended. **(B)** Rear ankle with the knee flexed. **(C)** Front ankle. **(D)** Ankle during deep squat sitting. Ankle dorsiflexion was measured using a goniometer as the angle between a line perpendicular to the floor and a line connecting the fibular head and lateral malleolus.

Weight-bearing ankle dorsiflexion ROM was measured using four methods: measuring the rear ankle with the knee extended (17) and flexed during forward lunge (18), measuring the front ankle during forward lunge (19), and measuring the ankle during deep squat sitting (20) (Figure 1). For the rear ankle measurements, the participants were instructed to step forward with the leg on the non-measurement side and to lean their lower leg forward as far as possible with the knee on the measurement side extended or flexed (Figures 1A,B). For the front ankle measurement, the participants were instructed to step forward with the lower leg being measured and lean their lower leg as far forward as possible in a forward lunge position against the wall (Figure 1C). The examiner instructed the participants to place their upper limbs against the wall for balance. Deep squat sitting measurements were performed as follows: participants were instructed to squat to the deepest position they could maintain for 3 s and then lean their lower leg forward as far as possible. They were instructed to stand with their feet shoulder-width apart, eyes looking straight ahead, and arms extended in front, parallel to the floor (Figure 1D). The participants were instructed to keep their heels on the ground during the tasks. The weight-bearing ankle dorsiflexion angle was measured using a goniometer to determine the angle between a perpendicular line to the floor and a line connecting the fibular head and lateral malleolus with a minimum value of 1°. The measurements were repeated twice for each method to verify reliability.

### 2.6 Measurement of ankle strength

Ankle strength was measured using a Biodex 3 dynamometer (Biodex Medical Systems, Shirley, NY, USA) to determine the ankle plantar/dorsiflexion muscles. The participants' knees were bent 30° while seated. Straps were used to stabilize the lower trunk, thigh, and ankle muscles. Ankle plantar/dorsiflexion measurements were made bilaterally isokinetically (concentric/concentric), and 2 sets of 5 maximal dynamic repeats were performed at an angular velocity of 60 °/s, separated by 30 s (20). Participants were positioned with their feet parallel to the floor to prevent hamstring strain. Finally, the peak torque/body weight ratio was computed after the torque was measured at a minimum of 1 Nm.

### 2.7 Measurement of gait parameters

Gait parameters were measured using a motion analyzer (MA-3000; Anima Corporation, Tokyo, Japan). Ten cameras were used with the motion analyzer, and data were acquired at 100 Hz. Each data point was low-pass filtered at a frequency of 10 Hz. The reflex marker sticking sites were the bilateral anterior superior iliac spine, greater trochanter, lateral femoral epicondyle, lateral malleolus, head of the fifth metatarsal bone, and midpoints of the left and right superior posterior iliac spines. Participants walked barefoot on a 6-m gait path at a self-selected speed. During walking, none of the participants required any walking aid. The gait parameters measured were the gait speed, cadence, step length, and plantar dorsiflexion angle of the ankle during the stance phase. In addition, sagittal plane knee angles were analyzed, with particular attention to the knee extension angle during the terminal stance phase, in order to evaluate potential compensatory movement patterns caused by limited ankle dorsiflexion. Gait was performed eight times, and the gait parameters were averaged using the motion analyzer's built-in software and analyzed.

### 2.8 Statistical analyses

G*Power 3.1 (Heinrich Hain University, Dusseldorf, Germany) (21) was used to calculate the required sample size (effect size *f*^2^ = 0.35, alpha error = 0.05, power = 0.80), and the result was 31. This study included 32 participants. The Shapiro–Wilk test was used to assess the data distribution. Means and standard deviations were calculated for normally distributed data, and medians and interquartile ranges were calculated for non-normally distributed data. The intraclass correlation coefficient (ICC) and Bland–Altman plot were used to evaluate the reliability of the weight-bearing ankle dorsiflexion ROM measurements. Using the first and second measurements, we calculated the ICC (1,1) and performed a Bland–Altman analysis. To evaluate the intrarater reliability, the ICC (1,1), standard error of measurement (SEM) (22), 95% confidence interval of the minimal detectable change (MDC_95_) (23), and relative repeatability (RR) (24) were calculated. The SEM, MDC_95_, and RR were calculated according to previous studies using the following formula: SEM = standard deviation × √(1−ICC), MDC_95_ = 1.96 × SEM × √2, and RR = MDC_95_/mean, respectively. Pearson's product–moment and Spearman's rank correlation coefficients were calculated to determine the correlation between the four SAFE-Q subscales and the measurement items after surgery, and a heat map was created. Finally, to clarify the factors related to postoperative QOL, a multiple regression analysis using the stepwise method was performed. The four subscales of the SAFE-Q (pain and pain-related, physical functioning and daily living, social functioning, and general health and well-being) were used as dependent variables, while the measurement items along with age, BMI, and the number of fractured malleoli were entered as independent variables.

Statistical significance was set at *P* < 0.05. All statistical analyses were performed using SPSS Statistics version 30 (IBM Corp., Armonk, NY, USA).

## 3 Results

### 3.1 Participant characteristics

Overall, 43 patients underwent ORIF during the study period. Of these, 11 were excluded (multiple fractures, *n* = 2; open fractures, *n* = 1; postoperative infection, *n* = 1; history of neurological disease, *n* = 1; refused to undergo postoperative measurements, *n* = 2; went to other hospitals, *n* = 4). Finally, 32 participants met the eligibility criteria and were included in this study. The participants' characteristics are summarized in Table 1. The median age of the participants was 48.5 (interquartile range, 23.5–61.5) years. The mean duration of fixation and crutch use after ORIF were 19.0 ± 12.3 and 49.3 ± 14.3 days, respectively. The mean time from ORIF to measurement was 92.0 ± 5.6 days.

**Table 1:** Participant characteristics.

Parameters	*n* = 32
Age (years)	48.5 (23.5–61.5)
Sex (male/female)	15/17
Height (m)	1.60 ± 0.08
Weight (kg)	56.7 (51.1–70.5)
Body mass index (kg/m^2^)	22.6 (19.7–26.9)
number of fractures (I/II/III)	15/6/11
Lauge–Hansen classification (SER/PER/SA)	19/4/9

Values are presented as median (interquartile range), mean ± standard deviation, or n/n. SER, supination–external rotation; PER, pronation–external rotation; SA, supination–adduction.

### 3.2 Participant measured values

The measured values of the participants are summarized in Table 2. The SAFE-Q scores of the four subscales (pain and pain-related, physical functioning and daily living, social functioning, and general health and well-being) were 75.6 ± 14.2, 74.1 ± 19.4, 69.9 ± 28.8, and 77.2 ± 23.2 points, respectively. The mean ankle dorsiflexion ROMs measured on the rear ankle with the knee extended during forward lunge, rear ankle with the knee flexed, front ankle during a forward lunge, and ankle during deep squat sitting were 15.6 ± 2.4, 18.3 ± 2.9, 21.8 ± 4.4, and 23.7 ± 5.0 degrees, respectively. Additionally, the knee extension angle during terminal stance phase was −3.1 ± 4.4 degrees.

**Table 2:** Participants' measured values.

Parameters	*n* = 32
SAFE-Q subscales score (points)	
Pain and pain-related	75.6 ± 14.2
Physical functioning and daily living	74.1 ± 19.4
Social functioning	69.9 ± 28.8
General health and well-being	77.2 ± 23.2
Non-weight-bearing ankle range of motion (°)	
Dorsiflexion with knee extended	13.3 ± 2.2
Dorsiflexion with knee flexed	17.5 ± 2.8
Plantarflexion	60.0 (55.0–61.3)
Weight-bearing ankle dorsiflexion range of motion (°)	
Rear ankle with the knee extended	15.6 ± 2.4
Rear ankle with the knee flexed	18.3 ± 2.9
Front ankle	21.8 ± 4.4
Deep squat sitting	23.7 ± 5.0
Ankle strength (Nm/kg)	
Plantarflexion	0.4 ± 0.2
Dorsiflexion	0.3 (0.2–0.4)
Gait parameters	
Gait speed (m/s)	0.93 ± 0.25
Step length (m)	0.53 ± 0.11
Cadence (step/min)	104.08 ± 10.19
Plantar dorsiflexion angle of the ankle during the stance phase (°)	24.2 ± 5.1
Knee extension angle during terminal stance phase (°)	−3.1 ± 4.4

Values are presented as mean ± standard deviation or median (interquartile range). SAFE-Q, Self-Administered Foot Evaluation Questionnaire.

### 3.3 Intrarater reliability of the weight-bearing ankle dorsiflexion ROM

The intrarater reliability values for weight-bearing ankle dorsiflexion ROM are summarized in Table 3. None of the methods showed fixed or proportional bias, and there was good agreement between the first and second measurements. The ICC (1,1) values for ankle dorsiflexion ROM measured on the rear ankle with the knee extended during a forward lunge, rear ankle with the knee flexed during a forward lunge, front ankle during a forward lunge, and ankle during deep squat sitting were 0.961, 0.959, 0.984, and 0.986, respectively. The SEMs were 0.49, 0.61, 0.54, and 0.58 degrees; the MDC_95_ values were 1.36, 1.68, 1.50, and 1.60 degrees; and the RRs were 0.09, 0.09, 0.07, and 0.07, respectively.

**Table 3:** Intrarater reliability of the weight-bearing ankle dorsiflexion range of motion.

Measurement tissue	Test 1	Test 2	ICC	95% CI	SEM	MDC_95_	RR
Rear ankle with the knee extended (°)	15.6	15.5	0.961	0.923–0.981	0.49	1.36	0.09
Rear ankle with the knee flexed (°)	18.3	18.2	0.959	0.918–0.980	0.61	1.68	0.09
Front ankle (°)	21.8	22.0	0.984	0.968–0.992	0.54	1.50	0.07
Deep squat sitting (°)	23.7	23.6	0.986	0.973–0.993	0.58	1.60	0.07

ICC, intraclass correlation coefficient; 95% CI, 95% confidence interval; SEM, standard error of measurement; MDC_95_, 95% confidence interval of the minimum detectable change; RR, relative repeatability.

### 3.4 Correlation coefficients heat map

**Figure 2:**
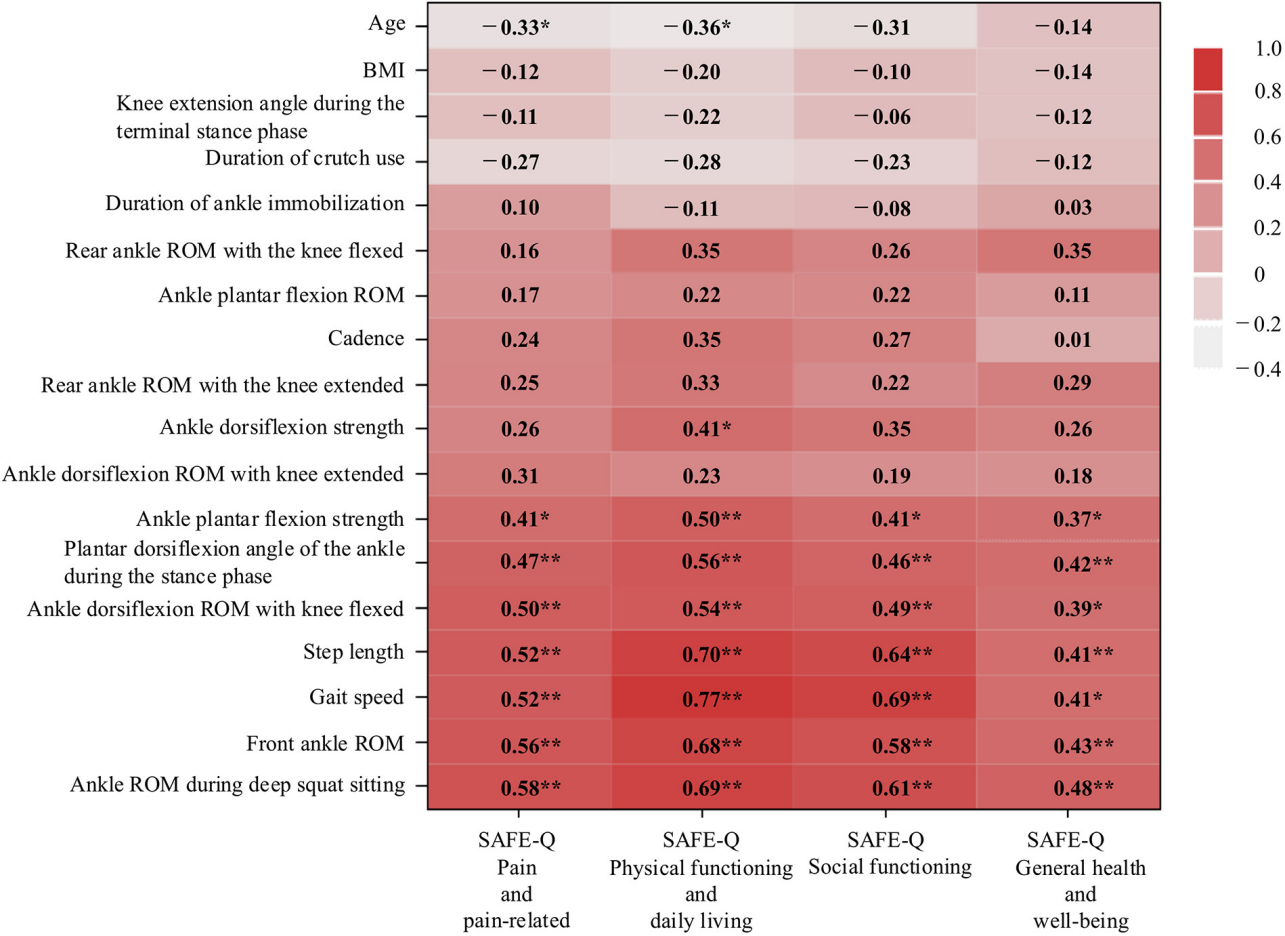
Heat map showing correlation coefficients between SAFE-Q subscales and measured values. Deep red pixels reflect a high positive correlation, whereas gray pixels reflect a negative correlation, and the numbers within the pixels indicate the correlation coefficient. Correlations were considered significant at *P < 0.05 and **P < 0.01. SAFE-Q, Self-Administered Foot Evaluation Questionnaire; BMI, body mass index; ROM, range of motion.

A heat map of the correlation coefficients between the SAFE-Q subscales and measured values is shown in Figure 2. Weight-bearing ankle dorsiflexion ROM during deep squat sitting positively correlated with the four subscales: pain and pain-related (*r* = 0.58; *P* < 0.001); physical functioning and daily living (*r* = 0.69; *P* < 0.001); social functioning (*r* = 0.61; *P* < 0.001); general health and well-being (*r* = 0.48; *P* = 0.005). Gait speed was also positively correlated with four subscales: pain and pain-related (*r* = 0.52; *P* = 0.002); physical functioning and daily living (*r* = 0.77; *P* < 0.001); social functioning (*r* = 0.69; *P* < 0.001); general health and well-being (*r* = 0.41; *P* = 0.002). In contrast, the knee extension angle during the terminal stance phase showed no significant correlation with any SAFE-Q subscales (*P* > 0.05).

### 3.5 Multiple regression analysis

The results of the multiple regression analysis are presented in Table 4. Weight-bearing ankle dorsiflexion ROM during deep squat sitting was selected as the independent variable for pain and pain-related [standardized partial regression coefficient (*β*) = 0.584, *P* < 0.001], physical functioning and daily living (*β* = 0.376; *P* = 0.006), social functioning (*β* = 0.317; *P* = 0.045), and general health and well-being (*β* = 0.483; *P* = 0.005). Furthermore, gait speed was selected as the independent variable for physical functioning and daily living (*β* = 0.555; *P* < 0.001) and social functioning (*β* = 0.514; *P* = 0.002). The adjusted coefficients of determination (*R*^2^) for pain and pain-related, physical functioning and daily living, social functioning, and general health and well-being as dependent variables were 0.319, 0.662, 0.517, and 0.207, respectively; the Durbin-Watson ratios were 1.666, 1.459, 2.143, and 1.640, respectively; the residuals were normally distributed, with *P* = 0.12, *P* = 0.10, *P* = 0.81, and *P* = 0.19, respectively.

**Table 4:** Multiple regression analysis.

Dependent variables	Independent variables	*B*	95% CI of *B*	*β*	*P* value	*R* ^2^	VIF
SAFE-Q							
Pain and pain-related	Ankle dorsiflexion ROM during deep squat sitting	1.671	0.805–2.538	0.584	<0.001	0.319	
Physical functioning and daily living	Gait speed	43.398	23.199–63.597	0.555	<0.001	0.662	1.465
	Ankle dorsiflexion ROM during deep squat sitting	1.469	0.459–2.479	0.376	0.006		
Social functioning	Gait speed	59.737	23.787–95.688	0.514	0.002	0.517	1.465
	Ankle dorsiflexion ROM during deep squat sitting	1.844	0.046–3.642	0.317	0.045		
General health and well-being	Ankle dorsiflexion ROM during deep squat sitting	2.255	0.730–3.781	0.483	0.005	0.207	

*B*, partial regression coefficient; CI, confidence interval; *β*, standardized partial regression coefficient; *R*^2^, adjusted coefficient of determination; VIF, variance inflation factor; SAFE-Q, self-administered foot evaluation questionnaire; ROM, range of motion.

## 4 Discussion

This study clarified the factors associated with QOL after ankle fracture surgery. The results support the hypothesis that weight-bearing ankle dorsiflexion ROM, particularly during deep squat sitting, is related to postoperative QOL. To our knowledge, this is the first study to demonstrate the ability of ankle dorsiflexion ROM during deep squat sitting to independently predict multiple domains of patient-reported QOL after ankle fracture surgery. This finding expands upon previous literature by showing that dorsiflexion under culturally and biomechanically relevant, high-demand conditions may offer more clinically meaningful insights than general weight-bearing or non-weight-bearing ROM, muscle strength, or gait parameter assessments.

Patient-reported outcome measures were used to bridge the gap between patients' subjective assessments and healthcare professionals' objective assessments of treatment effectiveness. Patient-reported outcome measures related to the ankle include the Olerud–Molander Ankle Score (25) and the Foot and Ankle Ability Measure (26); however, most of these assessments were developed in Europe or the United States, making it difficult to apply them to Asian lifestyles. Therefore, we evaluated the QOL using the SAFE-Q, which reflects the Asian lifestyle, and examined its relationship with physical function. The results suggested that weight-bearing ankle dorsiflexion ROM measured using various methods was more strongly associated with QOL than non-weight-bearing ROM after ankle fracture surgery. Hancock et al. (27) revealed that ankle dorsiflexion ROM affects the Olerud–Molander Ankle Score; however, only ROM without weight-bearing was measured, and ROM during weight-bearing was not considered. In daily life, most movements are performed in a weight-bearing position, such as walking and descending stairs, and a larger ankle dorsiflexion ROM is required than when not weighted. Previous studies have reported that gait and stair descent require ankle dorsiflexion ROMs of 10° and 21.1°, respectively, of the rear ankle (28, 29). Therefore, the weight-bearing ankle dorsiflexion ROM may be more strongly correlated than the non-weight-bearing ankle dorsiflexion ROM. Furthermore, multiple regression analysis showed that ankle dorsiflexion ROM during deep squat sitting affected the QOL. Deep squat sitting involves flexion of the entire body and is commonly performed in Asian countries in daily life, agricultural work, factory work, etc. (30, 31). Deep squat sitting becomes difficult after ankle fracture (32), which may have a negative impact on employment and return to work. Many patients with ankle fractures are of working age (33, 34), and the average age of the participants in this study was 48.5 years. Additionally, the ankle dorsiflexion ROM during deep squat sitting is approximately 28° (29), which requires a greater ROM than that of gait or stair descent. Therefore, weight-bearing ROM in a posture that requires a larger ankle dorsiflexion ROM may strongly affect postoperative QOL.

Multiple regression analysis showed that postoperative gait speed also affected the QOL. Previous studies have reported that gait speed influences quality of life (QOL) in older adults (7) and patients with stroke (35). However, only a limited number of studies have focused on this relationship in patients after ankle fracture surgery. By addressing this understudied population, the present study adds valuable insights to the literature. Hsu et al. (36) reported that gait speed was decreased even 4 months after ankle fracture surgery, and Wang et al. (10) reported that gait speed was decreased even 1 year after surgery. Gait is directly related to daily activities, such as housework, shopping, and traveling. Therefore, a decrease in gait speed, an aspect of walking ability, may indicate a decrease in the QOL. Additionally, younger patients may need to walk at the same speed as before the injury when going outdoors and returning to work. A decrease in walking speed may lead to reduced social outings and participation (37). Fung and Hays (38) reported that social participation is related to QOL and patient satisfaction. Therefore, a decrease in gait speed may reduce social participation and affect the QOL.

Clinically, the QOL after ankle fracture surgery is affected by weight-bearing ankle dorsiflexion ROM and gait speed. In particular, when evaluating weight-bearing ankle dorsiflexion ROM, it is necessary to measure dorsiflexion during deep squat sitting and lunge tests. Therefore, evaluation of these factors and early intervention to improve them may be important. The observed ROM limitations may arise from various sources, including joint stiffness caused by intra-articular adhesions or capsular tightness, as well as soft tissue restrictions such as shortened or tight calf muscles. Differentiating these causes is essential, as each has distinct implications for rehabilitation. For example, joint-related restrictions may respond better to mobilization techniques, while muscle tightness may be more effectively addressed with targeted stretching protocols. Our findings may also inform treatment programs to improve QOL after ankle fractures and provide the theoretical background needed to develop new treatment techniques. We also examined the knee extension angle during the terminal stance phase as a supplementary gait parameter to explore potential compensatory strategies resulting from limited ankle dorsiflexion. Although no significant correlation with QOL was observed in our sample, this parameter may still reflect important biomechanical adaptations. Future research involving larger cohorts and comprehensive gait analyses is warranted to clarify the role of sagittal plane knee kinematics in functional recovery and patient-reported outcomes after ankle fracture.

This study has some limitations. First, the gait parameters were measured barefoot. Wearing shoes may change the gait parameters (39). Therefore, there may be discrepancies when applying the results of this study to outdoor gait. Second, the amount of weight applied was not measured during ankle dorsiflexion ROM assessments in forward lunging and deep squat sitting. It should be noted that the amount of weight borne during these weight-bearing ROM assessments was not standardized or quantified, which may have introduced variability in the measurements. This may have contributed to inconsistency and should be addressed in future by more strictly controlling loading conditions. Third, the relatively small sample size, although determined by an *a priori* power analysis, may limit the external validity and generalizability of the findings. Caution is warranted when applying these results to broader patient populations. Finally, the follow-up period was limited to 3 months after surgery. Although this time point is clinically meaningful and captures key early functional improvements (40), longer-term changes in QOL and functional outcomes were not assessed. We are currently considering follow-up studies with extended observation periods to better understand the longitudinal impact of these physical factors on QOL. Considering these points, further research is needed.

In conclusion, ankle dorsiflexion ROM during deep squat sitting and gait speed were associated with the QOL of patients after ankle fracture surgery. Clinically, assessment of ankle dorsiflexion ROM and gait speed may be useful as part of a broader evaluation; however, further research is needed to confirm their influence on meaningful improvements in the QOL.

## Data Availability

The original contributions presented in the study are included in the article/Supplementary Material, further inquiries can be directed to the corresponding author.
